# Significance of the cribriform morphology area ratio for biochemical recurrence in Gleason score 4 + 4 prostate cancer patients following robot‐assisted radical prostatectomy

**DOI:** 10.1002/cam4.7086

**Published:** 2024-03-13

**Authors:** Kenji Shimodaira, Rie Inoue, Takeshi Hashimoto, Naoya Satake, Toshihide Shishido, Kazunori Namiki, Kazuharu Harada, Toshitaka Nagao, Yoshio Ohno

**Affiliations:** ^1^ Department of Urology Tokyo Medical University Tokyo Japan; ^2^ Anatomic Pathology Tokyo Medical University Tokyo Japan; ^3^ Department of Health Data Science Tokyo Medical University Tokyo Japan

**Keywords:** biochemical recurrence, cribriform morphology area ratio, Gleason pattern 4, prostate cancer, robot‐assisted radical prostatectomy

## Abstract

**Background:**

In prostate cancer, histological cribriform patterns are categorized as Gleason pattern 4, and recent studies have indicated that their size and percentage are associated with the risk of biochemical recurrence (BCR). However, these studies included a mixture of cases with various Gleason scores (GSs). We therefore examined the prognostic value of the area and percentage of cribriform patterns in patients with GS 4 + 4 prostate cancer.

**Methods:**

We investigated 108 patients with GS 4 + 4 prostate cancer who underwent robot‐assisted radical prostatectomy (RARP). After digitally scanning the hematoxylin and eosin‐stained slides, we measured the area of the entire cancer and cribriform patterns. Predictive factors for BCR were explored using log‐rank test and Cox proportional hazard model analyses.

**Results:**

Sixty‐seven (62.0%) patients had a cribriform pattern in RARP specimens, and 32 (29.6%) experienced BCR. The median total cancer area, cribriform pattern area, and percentage of cribriform pattern area (% cribriform) were 427.70 mm^2^ (interquartile range [IQR], 171.65–688.53 mm^2^), 8.85 mm^2^ (IQR, 0–98.83 mm^2^), and 2.44% (IQR, 0%–33.70%), respectively. Univariate analyses showed that higher preoperative serum prostate‐specific antigen (PSA) levels, positive resection margins, advanced pathological T stage, extraprostatic extension, larger total cancer area, larger cribriform morphology area, and higher % cribriform values were significantly associated with BCR. A multivariate analysis demonstrated that the PSA level (hazard ratio [HR], 1.061; 95% confidence interval [CI], 1.011–1.113; *p* = 0.017) and % cribriform (HR, 1.018; 95% CI, 1.005–1.031; *p* = 0.005) were independent predictors of BCR.

**Conclusions:**

An increased % cribriform value was associated with BCR in patients with GS 4 + 4 prostate cancer following RARP.

## Graphical abstract

The percentage of cribriform pattern in Gleason score 4 + 4 prostate cancer patients following robot‐assisted radical prostatectomy was independent predictor of biochemical recurrence.

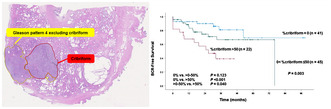

## 1 INTRODUCTION

In prostate cancer, the Gleason grading system is currently the most widely used histopathological grading system and is considered an extremely important prognostic factor. The definitions of Gleason patterns (GPs) have been modified over time.^1^, ^2^, ^3^ For example, the 2014 International Society of Urological Pathology (ISUP) consensus conference noted that all cribriform patterns, regardless of their morphology and size, were assigned to GP 4.

In addition to cribriform patterns, the GP 4 category includes three main architectural types: glomeruloid, poorly formed glands, and fused glands.^3^ The cribriform pattern is defined as a confluent sheet of contiguous malignant epithelial cells with multiple glandular lumina that are easily visible at low power (objective magnification ×10). There should be no intervening stroma or mucin separating the individual or fused glandular structures.^4^

The presence of a cribriform pattern in radical prostatectomy (RP) specimens has been independently associated with biochemical recurrence (BCR),^5^, ^6^, ^7^ distant metastasis,^6^ and disease‐specific death.^8^ Furthermore, recent studies have demonstrated that the size and percentage of cribriform patterns in GP 4 are associated with the risk of BCR.^9^, ^10^, ^11^ However, those studies included patients with prostate cancer who had a mixture of Gleason scores (GSs) 3 + 4 and 4 + 3.^5^, ^8^, ^9^, ^10^ To our knowledge, no previous report has investigated patients with GS 4 + 4 prostate cancer.

In the present study, we examined the association between the area and percentage of cribriform patterns and BCR in patients with GS 4 + 4 prostate cancer who underwent robot‐assisted RP (RARP).

## 2 PATIENTS AND METHODS

### 2.1 Patients and data collection

This retrospective study was conducted at Tokyo Medical University in Tokyo, Japan, and was approved by the Ethics Committee of Tokyo Medical University (No. T2020‐0429).

Patients without distant metastasis at the clinical stage were eligible for RARP. We reviewed specimens from all 1870 cases that underwent RARP at Tokyo Medical University from June 2006 to December 2018. Of these cases, only those with a final pathology diagnosis of GS 4 + 4 after reassessment of the Gleason score according to the 2014 ISUP criteria were selected.^3^

Patients who received neoadjuvant therapy or adjuvant therapy before BCR, had no histologic slides available, or were not followed postoperatively were excluded from the study. In addition, intraductal carcinoma (IDC) was histologically not included in GP 4 by the recommendations of the 2019 Genitourinary Pathology Society (GUSP).^12^ Therefore, we strictly excluded cases with IDC components based on a careful histomorphological observation using only hematoxylin and eosin (H&E)‐stained specimens according to the diagnostic criteria of the WHO Classification of Tumors of the Urinary System and Male Genital Organs.^13^ If GP 5 was found in less than 5% of all cancers, it was included as tertiary pattern.^14^ However, since the purpose of this study was to examine the proportion of cribriform morphology in GP 4, tertiary pattern 5 was excluded from the calculation of the total cancer area.

We ultimately analyzed 108 patients who underwent RARP and were pathologically diagnosed with GS 4 + 4 prostate cancer.

### 2.2 Pathological evaluation and measurement of the cancer area

RP specimens were sampled according to standardized clinical and pathological protocols.^14^ Tissue‐marking dye was applied to determine the extraprostatic extension and resection margin (RM) of the prostate cancer. After formalin fixation, we made a horizontal section perpendicular to the posterior surface of the urethral mucosa with a thickness of approximately 5 mm using a step section. The bladder neck and prostate apex sides were examined by sagittal sections with the most distal ends equally spaced.

**FIGURE 1:**
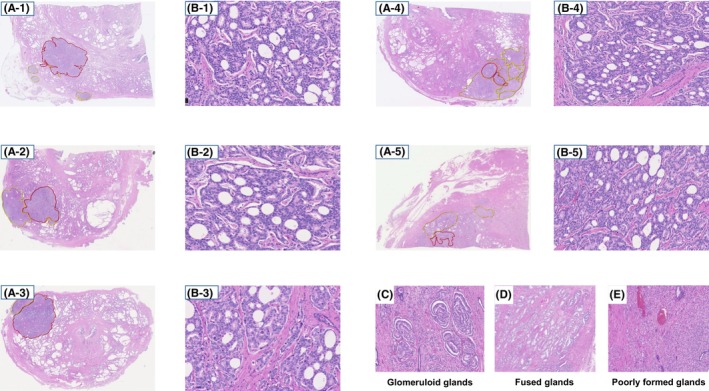
Ten virtual slides were annotated in this study. The slides of five cases are shown below. (A) A sample virtual slide after annotation of the histological cancer pattern (H&E, ×4). The area of cancer other than the cribriform pattern is enclosed within the yellow line, and the area of the cribriform pattern is indicated by the red line. (B) Magnified view of the cribriform architecture (H&E, ×100). (C–E) High power view of the non‐cribriform areas: glomeruloid (C), fused (D), and poorly formed glands (E) (H&E, ×100).

All prostate specimens obtained after RARP were diagnosed by two expert pathologists (R.I. and T.N.). All slides were digitally scanned (virtual slides) using a Nano Zoomer (Hamamatsu Photonics K.K., Hamamatsu, Japan). Figure 1 shows five sample slides with manually annotated cribriform and cancerous areas. Figure 1A shows the entire slide (H&E, ×4), and Figure 1B shows a magnified picture of the cribriform pattern of the corresponding slides in Figure 1A (H&E, ×100). In addition, non‐cribriform architectural types, including glomeruloid, fused, and poorly formed glands, are shown in Figures 1C–E, respectively (H&E, ×100). Each area was calculated digitally. The cribriform pattern had two forms: one interconnecting a large cribriform area or many non‐interconnecting small cribriform fields. However, we did not distinguish between the two forms. Furthermore, if many non‐interconnecting small cribriform patterns were observed, we encircled all small cribriform patterns and summed the marked areas as cribriform areas. All slides were examined to measure the total cancer and cribriform pattern areas. The percentage of the cribriform pattern area (% cribriform) was calculated as follows:

% cribriform = cribriform morphology area/total cancer area ×100.

### 2.3 Follow‐up

Prostate‐specific antigen (PSA) follow‐up was performed every 1–3 months following RARP, while BCR was defined as a rise in PSA levels ≥0.2 ng/ mL, twice consecutively. If the PSA level immediately after RARP was ≥0.2 ng/mL, the recurrence day was defined as the day of surgery.

### 2.4 Statistical analyses

The study endpoint was BCR. Correlations between continuous variables were analyzed using Spearman's rank correlation coefficients. Variables of the different groups were compared using the Mann–Whitney *U*‐test, and categorical parameters were analyzed using the chi‐squared test. BCR‐free survival curves were drawn using the Kaplan–Meier method, and the significance of differences in survival curves between groups was evaluated using the log‐rank test. Univariate and multivariate analyses were performed using the Cox regression model. The cribriform morphological area was not included in the multivariate analysis. The total cancer area, cribriform morphology area, and % cribriform are related, so knowing two of them, determines the third. Including all three in the Cox regression model can cause interpretation problems owing to multicollinearity.

To evaluate the degree of multicollinearity among the total cancer area, cribriform morphology area, and % cribriform, correlation coefficients were calculated. The cribriform morphology area was found to have the highest correlation with the other variables, with correlation coefficients of 0.946 and 0.316 for the total cancer area and % cribriform, respectively. By excluding the cribriform morphology area, these issues were avoided, and a clear, independent set of predictors, specifically cancer size and the proportion of the cribriform part, was created.

For all analyses, statistical significance was set at *p* < 0.05. Statistical analyses were performed using the SPSS version 27 software program (IBM Corp., Armonk, NY, USA).

## 3 RESULTS

**FIGURE 2:**
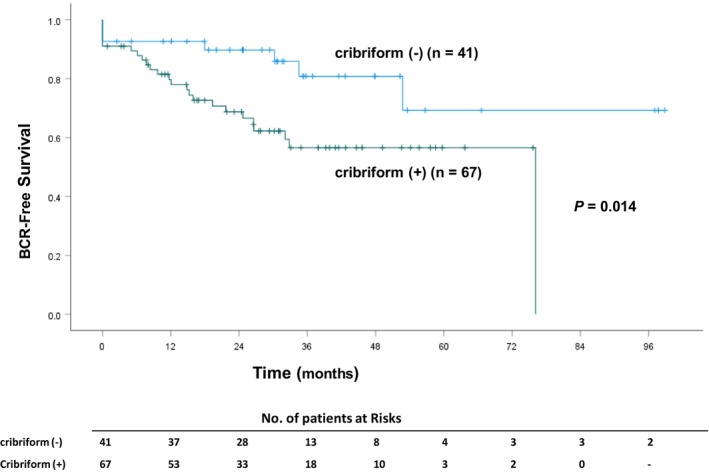
Kaplan–Meier BCR‐free survival curves according to the cribriform pattern presence in patients with Gleason score 4 + 4 prostate cancer.

The patient characteristics and pathological findings are shown in Table 1. The cribriform pattern was identified in 67 of 108 (62.0%) patients with GS 4 + 4 prostate cancer after RARP. Thirty‐two (29.6%) patients had experienced BCR, with a mean follow‐up of 36.5 months. The presence of a cribriform pattern was significantly associated with BCR. The 3‐year BCR‐free rate in patients with cribriform GP 4 was significantly lower than that in patients without cribriform GP 4 (56.3% vs. 80.3%; *p* = 0.014; Figure 2).

**TABLE 1:** Patient characteristics and clinicopathological findings.

		Cribriform		*p‐*value
		No	Yes	
Age (years)	66.4 (62.8–70.3)	65.8 (66.0, 61.0–70.0)	66.7 (66.0, 63.5–71.0)	^a^0.346
PSA (ng/mL)	10.59 (5.68–11.80)	9.07 (7.60, 5.60–10.40)	11.52 (8.60, 5.80–13.05)	^a^0.199
Clinical findings				
Clinical T stage				
T1c	40 (37.0)	15 (13.9)	25 (23.1)	^b^0.190
T2a	32 (29.6)	12 (11.1)	20 (18.5)	
T2b	12 (11.1)	5 (4.6)	7 (6.5)	
T2c	11 (10.2)	4 (3.7)	7 (6.5)	
T3a	7 (6.5)	2 (1.9)	5 (4.6)	
T3b	6 (5.6)	3 (2.8)	3 (2.8)	
Pathological findings				
Pathological T				
2	67 (62.0)	27 (25.0)	40 (37.0)	^b^0.523
3	41 (38.0)	14 (13.0)	27 (25.0)	
RM				
0	66 (61.1)	25 (23.1)	41 (38.0)	^b^0.982
1	42 (38.9)	16 (14.8)	26 (24.1)	
Lymphatic invasion				
0	74 (68.5)	29 (26.9)	45 (41.7)	^b^0.698
1	34 (31.5)	12 (11.1)	22 (20.4)	
Vascular invasion				
0	67 (62.0)	26 (24.1)	41 (38.0)	^b^0.817
1	41 (38.0)	15 (13.9)	26 (24.1)	
Perineural invasion				
0	17 (15.7)	7 (6.5)	10 (9.3)	^b^0.766
1	91 (84.3)	34 (31.5)	57 (52.8)	
Extraprostatic extension				
0	58 (53.7)	23 (21.3)	35 (32.4)	^b^0.696
1	39 (36.1)	13 (12.0)	26 (24.1)	
*x*	11 (10.2)	5 (4.6)	6 (5.6)	
Seminal vesicle invasion				
0	93 (86.1)	36 (33.3)	57 (52.8)	^b^0.691
1	15 (13.9)	5 (4.6)	10 (9.3)	
Tertiary 5				
0	76 (70.4)	33 (30.6)	43 (39.8)	^b^0.072
1	32 (29.6)	8 (7.4)	24 (22.2)	
Follow‐up period (month)	36.5 (22.0–47.3)	39.3 (34.0, 24.0–51.0)	34.8 (30.0, 19.0–45.0)	^a^0.355
BCR				
Yes	32 (29.6)	7 (6.5)	25 (23.1)	^b^0.025
No	76 (70.4)	34 (31.5)	42 (38.9)	

*Note*: Mean (median, interquartile) or *n* (%).

Abbreviations: BCR, biochemical recurrence; GS, Gleason score; PSA, prostate‐specific antigen; RM, resection margin.

^a^ Mann–Whitney test.

^b^ Chi‐squared test.

A total of 34 patients underwent a prostate needle biopsy before RARP at our institution (Table 2). Of these, 25 (73.5%) showed negative cribriform morphology on a needle biopsy. Thirteen (38.2%) were true negatives, while 9 (26.5%) had a positive result for cribriform morphology on a needle biopsy (true positive). All cases with a % cribriform >50% showed multiple positive cribriform cores.

**TABLE 2:** Prostate needle biopsy and postoperative pathological findings in our institution (*n* = 34).

			Results of prostate needle biopsy				RARP		
			Total number of the cores	Number of the positive cores	Percent of the positive cores	Target biopsy	% cribriform = 0 (*n* = 13)	0 < % cribriform ≤50 (*n* = 16)	% cribriform >50 (*n* = 5)
Prostate biopsy	GS	4 + 3	13.50 (13.50, 13.25–13.75)	7.5 (7.5, 4.75–10.25)	54.12 (54.12, 34.75–73.49)	0 (0%)	1 (2.9%)	0 (0.0%)	1 (2.9%)
		4 + 4	14.30 (12.00, 12.00–14.00)	3.90 (3.50, 2.25–5.00)	28.55 (25.00, 17.50–33.33)	11 (32.4%)	11 (32.4%)	15 (44.1%)	4 (11.8%)
		4 + 5	15.00 (15.00, 15.00–15.00)	5.00 (5.00, 5.00–5.00)	33.33 (33.33, 33.33–33.33)	1 (2.9%)	0 (0.0%)	1 (2.9%)	0 (0.0%)
		5 + 4	14.00 (14.00, 14.00–14.00)	4.00 (4.00, 4.00–4.00)	28.57 (28.57, 28.57–28.57)	1 (2.9%)	1 (2.9%)	0 (0.0%)	0 (0.0%)
	Cribriform	(−)					13 (38.2%)	10 (29.4%)	2 (5.9%)
		(+)				Single core	0 (0.0%)	3 (8.8%)	0 (0.0%)
						Multiple cores	0 (0.0%)	3 (8.8%)	3 (8.8%)

*Note*: Mean (median, interquartile) or *n* (%).

Abbreviations: % cribriform, percent of cribriform area/total cancer area; GS, Gleason score; RARP, robot‐assisted radical prostatectomy.

The digital imaging analysis revealed that the median total cancer area and cribriform pattern area were 427.70 mm^2^ (interquartile range [IQR], 171.65–688.53 mm^2^) and 8.85 mm^2^ (IQR, 0–98.83 mm^2^), respectively, and the median % cribriform was 2.44% (IQR, 0%–33.70%) (Table 3). The associations among the total cancer area, cribriform pattern area, % cribriform, and clinicopathological parameters are shown in Table 4. The preoperative PSA level, pathological T stage, RM, lymphatic invasion, perineural invasion, extraprostatic extension, seminal vesicle invasion, and presence of tertiary 5 were associated with the total cancer area. The pathological T stage was associated with cribriform morphology area, but other factors were not. No association was found between the % cribriform and clinicopathological parameters.

**TABLE 3:** Measurement outcome.

	Median (IQR)
Total cancer area (mm^2^)	427.70 (171.65–688.53)
Cribriform morphology area (mm^2^)	8.85 (0–98.83)
% cribriform	2.44 (0–33.70)

Abbreviations: % cribriform, percent of cribriform area/total cancer area; IQR, interquartile range.

**TABLE 4:** Results of relationship among cribriform area, % cribriform, and other clinicopathological factors.

Variables	Total cancer area	Cribriform morphology area	% cribriform
	*p*‐value	*p*‐value	*p*‐value
Age	^a^0.501	^a^0.231	^a^0.318
PSA	^a^ < 0.001	^a^0.078	^a^0.728
Pathological T	^b^ < 0.001	^b^0.033	^b^0.367
2	321.89 (239.70, 127.05–501.30)	48.37 (7.00, 0–66.90)	17.17 (2.07, 0–20.13)
3	1008.54 (707.70, 512.30–1205.20)	283.75 (27.30, 0–246.50)	22.92 (2.56, 0–50.66)
RM	^b^ < 0.001	^b^0.222	^b^0.881
0	342.68 (247.80, 126.58–520.83)	54.85 (7.60, 0–67.45)	18.75 (2.44, 0–29.59)
1	959.53 (707.35, 365.18–947.43)	267.97 (15.10, 0–227.30)	20.30 (2.41, 0–49.02)
Lymphatic invasion	^b^ < 0.001	^b^0.101	^b^0.418
0	456.16 (300.00, 126.58–567.93)	102.12 (7.30, 0–68.43)	17.72 (1.92, 0–30.33)
1	857.69 (699.50, 252.13–1175.50)	215.24 (22.45, 0–289.33)	22.89 (4.99, 0–47.68)
Vascular invasion	^b^0.374	^b^0.192	^b^0.374
0	501.15 (380.10, 166.45–632.95)	100.15 (7.20, 0–66.90)	17.59 (1.67, 0–29.17)
1	715.61 (484.10, 172.00–765.10)	199.14 (23.40, 0–111.40)	22.23 (7.43, 0–37.20)
Perineural invasion	^b^0.004	^b^0.179	^b^0.512
0	258.55 (128.00, 75.80–438.40)	31.65 (4.90, 0–21.20)	12.52 (2.56, 0–8.76)
1	643.10 (178.50, 199.70–718.40)	157.55 (10.00, 0–102.15)	20.63 (2.27, 0–37.96)
Extraprostatic extension	^b^ < 0.001	^b^0.153	^b^0.924
0	293.50 (233.10, 120.23–476.95)	53.65 (7.1, 0–61.43)	19.50 (2.44, 0–29.59)
1	917.88689.45, 332.35–947.43)	235.26 (15.95, 0–159.33)	19.18 (2.41, 0–38.34)
Seminal vesicle invasion	^b^ < 0.001	^b^0.490	^b^0.924
0	476.56 (304.50, 130.70–615.00)	107.66 (8.80, 0–74.90)	19.52 (2.56, 0–33.62)
1	1239.81 (764.20, 625.10–1278.25)	324.19 (12.60, 0–145.45)	18.27 (1.16, 0–32.94)
Tertiary 5	^b^0.001	^b^0.151	^b^0.387
0	535.00 (277.15, 123.13–600.30)	140.15 (7.10, 0–99.43)	19.28 (2.44, 0–34.75)
1	695.53 (607.25, 366.63–864.55)	131.98 (18.85, 2.18–84.83)	19.51 (3.26, 0.42–23.52)

*Note*: Mean (median, interquartile) or *n* (%).

Abbreviations: % cribriform, percent of cribriform area/total cancer area; PSA, prostate‐specific antigen; RM, resection margin.

^a^ Spearman's rank correlation coefficient.

^b^ Mann–Whitney test.

The results of the Cox regression analysis are presented in Table 5 and Table S1. Univariate analyses showed that higher preoperative serum PSA levels, positive RM, an advanced pathological T stage, extraprostatic extension, a larger total cancer area, a larger cribriform morphology area, and a higher percentage of cribriform lesions were significantly associated with BCR. A multivariate analysis demonstrated that PSA (hazard ratio [HR], 1.061; 95% confidence interval [CI], 1.011–1.113; *p* = 0.017) and % cribriform (HR, 1.018; 95% CI, 1.005–1.031; *p* = 0.005) were independent predictors of BCR.

**TABLE 5:** Result of univariate and multivariate analyses.

Parameter	Univariate		Multivariate					
			Model 1		Model 2		Model 3	
	HR (95%CI)	*p*‐value	HR (95%CI)	*p*‐value	HR (95%CI)	*p*‐value	HR (95%CI)	*p‐*value
Age (years) (continuous)	0.966 (0.908–1.029)	0.285	0.971 (0.915–1.030)	0.322	0.984 (0.924–1.047)	0.607	0.989 (0.922–1.060)	0.748
PSA (ng/mL) (continuous)	1.065 (1.027–1.105)	<0.001	1.074 (1.035–1.114)	<0.001	1.064 (1.022–1.107)	0.002	1.061 (1.011–1.113)	0.017
RM	3.215 (1.575–6.561)	0.001	‐		2.556 (0.990–6.603)	0.053	2.505 (0.939–6.683)	0.067
Pathological T (2 vs. 3)	2.100 (1.042–4.231)	0.038	‐		1.231 (0.394–3.839)	0.721	0.959 (0.251–3.657)	0.951
Lymphatic invasion	1.384 (0.671–2.852)	0.379	‐		‐		0.755 (0.305–1.867)	0.543
Vascular invasion	1.365 (0.681–2.736)	0.38	‐		‐		1.235 (0.573–2.661)	0.59
Perineural invasion	1.720 (0.599–4.938)	0.313	‐		‐		1.629 (0.464–5.725)	0.447
Extraprostatic extension	2.260 (1.100–4.645)	0.027	‐		0.907 (0.246–3.344)	0.884	0.923 (0.230–3.694)	0.909
Seminal vesicle invasion	1.651 (0.676–4.033)	0.271	‐		‐		1.298 (0.451–3.740)	0.629
Tertiary5	1.288 (0.606–2.737)	0.51	‐		1.263 (0.564–2.832)	0.57	1.208 (0.524–2.785)	0.657
Total cancer area (mm^2^) (continuous)	1.001 (1.000–1.001)	<0.001	‐		‐		1.000 (1.000–1.001)	0.303
Cribriform morphology area (mm^2^) (continuous)	1.001 (1.001–1.002)	<0.001	‐		‐		‐	
% cribriform (%) (continuous)	1.015 (1.005–1.026)	0.004	1.019 (1.008–1.030)	<0.001	1.019 (1.007–1.031)	0.002	1.018 (1.005–1.031)	0.005

Abbreviations: % cribriform, percent of cribriform area/total cancer area; HR, Hazard ratio; PSA, prostate‐specific antigen; RM, resection margin.

**FIGURE 3:**
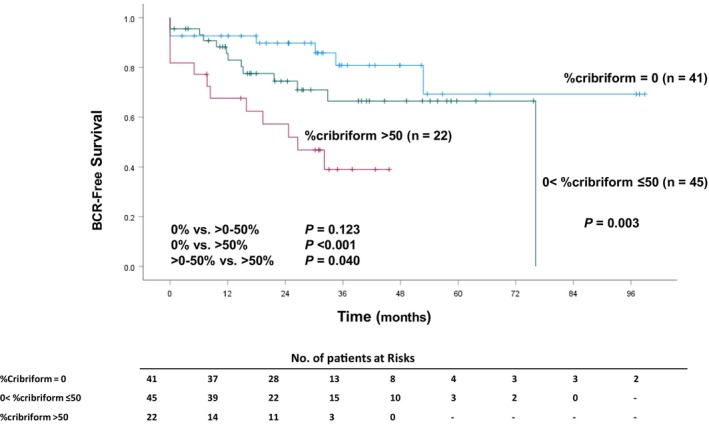
Kaplan–Meier BCR‐free survival curves according to the % cribriform in patients with Gleason score 4 + 4 prostate cancer.

As reported previously,^15^ the % cribriform was categorized by setting effectual cutoff values. The best discriminating cutoff point, or the value that had the most significant *p*‐value on the log‐rank test, was determined by testing all possible cutoff points within the central 80% of the distribution of values. All cutoff points were rounded to clinically relevant or convenient values, and the % cribriform values were categorized into three groups (0% vs. >0% to 50% vs. >50%). The 3‐year BCR‐free rates were 80.3% in the 0% group, 62.7% in the >0% to 50% group, and 39.1% in the >50% group, and there was a statistically significant difference among the groups (*p* = 0.003, Figure 3).

## 4 DISCUSSION

In this study, we digitally measured the area of the total cancer and cribriform patterns. The total cancer area, cribriform pattern area, and % cribriform were significantly associated with BCR after RARP. In the multivariate analysis, we demonstrated that the preoperative PSA level and % cribriform were independent predictors of BCR in patients with GS 4 + 4 at RARP.

Many studies thus far have suggested that the presence of cribriform patterns in RP specimens is associated with a poor prognosis.^5^, ^6^, ^7^, ^8^, ^9^, ^10^, ^11^, ^16^, ^17^

Among these studies, three evaluated the prognostic value of the size and percentage of the cribriform pattern.^9^, ^10^, ^11^ First, Iczkowski et al. conducted a case–control study to analyze the association between histologic patterns, including cribriform patterns, and BCR. In their study, cribriform patterns were divided into two categories (small cribriform pattern: medium‐sized acinar spaces with rounded contours, no solid foci, and ≤ 12 lumens; and large cribriform pattern: expansive cribriform to focally solid large acini with >12 lumens). They reported that the presence of both large and small cribriform patterns was significantly associated with BCR, and that BCR was associated with the cumulative area sum of cribriform cancer with an odds ratio of 1.173. Second, Chan et al. examined the quantitative cutoff for cribriform size following RP. They showed that a cribriform diameter >0.25 mm was significantly associated with BCR, independent of the preoperative PSA level, GS, extraprostatic extension, surgical margin positivity, or seminal vesicle invasion. The recurrence‐free survival‐based outcome identified >0.25 mm as the optimal size criterion for large cribriform. Finally, Hollemanns et al. studied the association between recurrence and cribriform tumor size in patients with ISUP grade 2 prostate cancer. Small and large cribriform patterns in their study were distinguished based on the diameter being at least twice the size of the adjacent preexisting normal glands. A large cribriform pattern was associated with a higher percentage of GP 4 and extraprostatic extensions. They reported that a large cribriform pattern was an independent predictor of BCR in patients with ISUP grade 2 prostate cancer. In all of these studies, larger cribriform patterns tended to be associated with BCR.

However, these three studies covered various GSs, such as GS 3 + 4, GS 4 + 3, and GS 4 + 4. It has been reported that the cribriform pattern is more common in GS 4 + 3 than in GS 3 + 4 cancers and that the percentage of GP 4 is associated with BCR.^5^ However, in patients with a mixture of various GSs, the impact of the area and percentage of the cribriform pattern as GP 4 on the clinical outcome may be affected by the percentage of GP components other than GP 4. Therefore, in the present study, we investigated the above‐mentioned factors only in GS 4 + 4 in RARP specimens, finding that % cribriform was an independent predictor of BCR in these patients. Furthermore, there was a significant difference in BCR‐free rates among the 3% cribriform groups (0%, >0% to 50%, and > 50%).

Several studies have demonstrated other independent risk factors associated with BCR after RP.^18^, ^19^ For example, Sasaki et al. evaluated a cohort of 298 patients treated with RARP among the grade group 4.^18^ They reported that seminal vesicle invasion (HR, 2.39; 95% CI, 1.18–4.83) and RM (HR, 2.07; 95% CI, 1.24–3.48) were significant independent risk factors associated with BCR. In addition, Gandaglia et al. also showed that seminal vesicle invasion (HR, 1.81; 95% CI, 1.47–2.32) and RM (HR, 1.23; 95% CI, 1.03–1.47) were important BCR factors in a cohort of 1089 patients with grade group 4 following RP.^19^ The HR values with seminal vesicle invasion and RM were shown to be 1.298 (95% CI, 0.451–3.740) and 2.505 (95% CI, 0.939–6.683), respectively, in their study.

The difference in HR values with seminal vesicle invasion and RM between this study and previous reports may be due to population differences. Previous studies have examined the BCR factor among grade group 4, whereas we did so only in GS 4 + 4 populations. The IDC of the prostate (IDC‐P) is an important poor prognostic factor.^20^, ^21^ Because of the histologic similarity between the cribriform pattern and IDC‐P, they may be confused, and indeed, some studies have examined the two together without distinguishing them. There are various diagnostic criteria for IDC‐P, and a consensus in this point has not yet been reached.^3^, ^13^, ^22^, ^23^ In addition, the 2019 GUPS recommends that IDC‐P should not be included in the final GS on RP.^12^ The main purpose of the present study was to determine the prognostic significance of the cribriform pattern of GP 4. Therefore, in accordance with the 2019 GUSP recommendations,^12^ we excluded cases with an IDC component.

Although this study provides important insights into the association between % cribriform and BCR in GS 4 + 4 patients who underwent RARP, it had several limitations. First, because this was a retrospective analysis of data collected from a single institution, the number of included cases was relatively small. Second, we did not obtain the disease‐specific or overall survival data because of the relatively short follow‐up period. Third, we did not consider the size of each cribriform pattern component, as previously reported, and a future study evaluating its prognostic impact is expected. Fourth, other architectural types with GP 4, such as glomeruloid, poorly formed, and fused glands, were not investigated in this study. Although the cribriform pattern was the most prevalent among the GP 4 architectures, a combination of more than two architectures has also been recognized in GP 4.^5^ The presence of a glomeruloid architecture has been reported to be associated with a reduced risk of BCR in contrast to the cribriform pattern.^5^ Further studies examining the prognostic value of the area and percentage of each GP 4 pattern in patients with GS 4 + 4 are warranted. Finally, we did not evaluate cribriform structures present in the intraductal spaces. This is because IDC‐P is an independent poor prognostic factor.^20^, ^21^ The IDC component is not part of the GP 4 assessment^12^ but is sometimes perceived as a cribriform GP 4 pattern.^22^ Thus, in the present study, we focused only on the cribriform pattern of GP 4 and excluded cases with IDC components. Although there are various definitions for the diagnosis of IDC‐P, including the use of ancillary testing by immunohistochemistry,^24^, ^25^ the evaluation was solely based on histomorphological observations of H&E‐stained specimens, in accordance with the diagnostic criteria of the WHO Classification of Tumors of the Urinary System and Male Genital Organs.^13^

Despite these limitations, our study is the first to demonstrate an association between the area and percentage of cribriform patterns and BCR. The 2014 ISUP consensus conference introduced a new grade group system in which GS 7 was divided into two grade groups (Group 2, GS 3 + 4; Group 3, GS 4 + 3),^3^ indicating that the proportion of GP 4 is important. Furthermore, a later ISUP consensus conference in 2019 recommended reporting the percentage of GP 4 for all GS 7 (Groups 2 and 3) in biopsies, along with the presence of a cribriform pattern.^26^ We hope that our data will be useful for future studies of prostate cancer grading systems.

## 5 CONCLUSION

This study demonstrated that the PSA level and % cribriform were independent predictors of BCR in patients with GS 4 + 4 following RARP. It is important to monitor the patient's postoperative course and consider adjuvant therapies, such as radiotherapy or hormonal therapy. Further studies are required to validate these findings.

## AUTHOR CONTRIBUTIONS

**Kenji Shimodaira:** Conceptualization (equal); data curation (equal); formal analysis (equal); investigation (equal); methodology (equal); project administration (equal); writing – original draft (equal). **Rie Inoue:** Investigation (supporting). **Takeshi Hashimoto:** Data curation (supporting). **Naoya Satake:** Data curation (supporting). **Toshihide Shishido:** Data curation (supporting). **Kazunori Namiki:** Data curation (supporting). **Kazuharu Harada:** Formal analysis (supporting). **Toshitaka Nagao:** Investigation (lead); project administration (lead); writing – review and editing (supporting). **Yoshio Ohno:** Data curation (supporting); investigation (lead); project administration (lead); writing – review and editing (lead).

## ETHICS STATEMENT

This study was approved by the Ethics Committee of the Tokyo Medical University (No. T2020‐0429). As this was a retrospective study, we explained the details of this study to the patients in a public letter. Therefore, the need for informed consent was waived by the Ethics Committee of Tokyo Medical University.

## Supporting information


**TableS1.** Result of univariate and multivariate analyses.

## Data Availability

The data that support the findings of this study are available from the corresponding author upon reasonable request.
